# Analysis of predictors of mortality and clinical outcomes of different subphenotypes for moderate-to-severe pediatric acute respiratory distress syndrome: A prospective single-center study

**DOI:** 10.3389/fped.2022.1019314

**Published:** 2022-11-01

**Authors:** Qingyue Wang, Yanling Liu, Yueqiang Fu, Chengjun Liu, Jing Li, Hongxing Dang

**Affiliations:** ^1^ Department of Pediatric Intensive Care Unit, Ministry of Education Key Laboratory of Child Development and Disorders, Children's Hospital of Chongqing Medical University, Chongqing, China; ^2^ National Clinical Research Center for Child Health and Disorders, China International Science and Technology Cooperation Base of Child Development and Critical Disorders, Chongqing, China; ^3^ Chongqing Key Laboratory of Child Health and Nutrition, Chongqing, China

**Keywords:** acute respiratory distress syndrome, mortality, predictors, intensive care unit, children, clinical phenotype

## Abstract

**Background:**

This study aimed to observe the prognosis of patients with moderate-to-severe pediatric acute respiratory distress syndrome (PARDS) admitted to the Pediatric Intensive Care Unit (PICU) as a function of underlying conditions and available treatment strategies, and to investigate the risk factors for death and the outcomes of different clinical subphenotypes.

**Methods:**

Patients were divided into non-survivors and survivors according to the prognosis 28 days after the diagnosis. The risk factors for death and the predictive value of relevant factors for mortality were analyzed. Latent class analysis was used to identify different clinical subphenotypes.

**Results:**

A total of 213 patients with moderate-to-severe PARDS were enrolled, of which 98 (46.0%) died. Higher PELOD2 scores (OR = 1.082, 95% CI 1.004–1.166, *p* < 0.05), greater organ failure (OR = 1.617, 95% CI 1.130–2.313, *p* < 0.05), sepsis (OR = 4.234, 95% CI 1.773–10.111, *p* < 0.05), any comorbidity (OR = 3.437, 95% CI 1.489–7.936, *p* < 0.05), and higher infiltration area grade (IAG) (OR = 1.980, 95% CI 1.028–3.813, *p* < 0.05) were associated with higher mortality. The combination of these five indicators had the largest area under the curve (sensitivity 89.79%, specificity 94.78%). Patients were classified into higher-risk and lower-risk phenotype group according to the latent class analysis. Compared to the lower-risk phenotype, more patients with higher-risk phenotype suffered from sepsis (24.40% vs. 12.20%, *p* < 0.05), inherited metabolic diseases (45.80% vs. 25.60%, *p* < 0.05), positive respiratory pathogens (48.10% vs. 26.80%, *p* < 0.05), and higher IAG (*p* < 0.05); they also had significantly higher PIM3 and PELOD2 scores (*p* < 0.05), with an extremely high mortality rate (61.1% vs. 22.0%, *p* < 0.05).

**Conclusions:**

Moderate-to-severe PARDS has high morbidity and mortality in PICU; a higher PELOD2 score, greater organ failure, sepsis, any comorbidity, and higher IAG were risk factors for death, and the combination of these five indicators had the greatest value in predicting prognosis. More patients with sepsis, positive respiratory pathogens, higher PIM3 and PELOD2 scores, and higher IAG were in higher-risk phenotype group, which had worse outcomes. Clear classification facilitates targeted treatment and prognosis determination.

## Introduction

Acute respiratory distress syndrome (ARDS) is a heterogeneous syndrome caused by multiple factors, with diffuse alveolar damage as the main pathologic hallmark (1). Although the pathogenesis and clinical management of pediatric acute respiratory distress syndrome (PARDS) have been extensively studied over the past 40 years, it remains a fatal disease in the pediatric intensive care unit (PICU) (2), with a higher mortality rate in patients with severe PARDS from middle-income countries (3, 4). There are still great challenges in the research of early diagnosis and treatment of PARDS, which is a main focus of pediatric critical care medicine.

Due to the lack of systematic studies with large sample sizes and the heterogeneity of pathogenesis and treatments of PARDS, the predictors of mortality of moderate-to-severe PARDS are still widely debated (5–7). Therefore, systematically analyzing the treatments and categorizing patients with moderate-to-severe PARDS can provide the possibility of early identification of prognostic factors, followed by implementation of targeted therapy and management and improved prognosis of moderate-to-severe PARDS (8).

Thus, we conducted a systematic prospective observational study of patients with moderate-to-severe PARDS in the PICU to understand the prognostic impact in terms of their underlying conditions and treatment strategies, to analyze the prognostic factors, and to explore the clinical outcomes of different subphenotypes. We hope to provide a basis for clinical work and maximize the prognosis of patients with moderate-to-severe PARDS.

## Materials and methods

### Participants

This prospective observational study was conducted in the PICU of the Children's Hospital of Chongqing Medical University, a tertiary teaching hospital in the Chinese National Clinical Medical Research Center, from September 2019 to March 2022. The study was approved by the ethics committee of our hospital, and informed consent requirements were waived. Patients were identified through electronic health records and by manual screening. All data were kept confidential in accordance with local regulations.

Inclusion criteria were as follows: all patients who met the diagnostic criteria of the Pediatric Acute Lung Injury Consensus Conference (PALICC) (9).

Exclusion criteria were as follows: patients without invasive mechanical ventilation; age ≤28 days; mild PARDS; lack of records; missed visits within 28 days after diagnosis.

### Data collected

Anonymous raw data from patients' medical records, including gender, age, weight, comorbidities, treatment information (mechanical ventilation, corticosteroids, neuromuscular blocking agents, surfactants, pulmonary vasodilators, blood transfusion, and prone ventilation), blood gas analysis on the first day of diagnosis of PARDS (9:00 a.m.), and pathogenic findings of blood and sputum were collected. The Pediatric Index of Mortality 3 score (PIM3 score) on admission, Pediatric Logistic Organ Dysfunction 2 score (PELOD2 score), and weight-for-age *z*-score when the patient was enrolled were calculated. PEEP titration protocol was used in the treatment of every participant in the study. We also defined the infiltration area grade (IAG) 1–4 from the chest radiographs of all patients on the day of diagnosis; a single quadrant was recorded as IAG-1, two quadrants were recorded as IAG-2, three quadrants were recorded as IAG-3, and four or more quadrants were recorded as IAG-4. The horizontal plane of the ipsilateral pulmonary artery at its midpoint at the hilum was used to distinguish between the upper and lower quadrant of a lung field. All included children were followed up until 28 days after diagnosis. Mortality, duration of invasive mechanical ventilation, length of ICU stay, and hospitalization time were all recorded.

### Statistical analyses

SPSS 26.0 was used for statistical analysis. Continuous variables with a normal distribution were tested using the *t*-test, expressed as the mean ± standard deviation, while those with a non-normal distribution were tested using the Mann–Whitney U-test, described as the median with the 25%–75% interquartile range. Categorical variables were compared by chi-squared test or Fisher exact test with counts and percentages. Univariate and multivariable analyses through binary logistic regressions were used to obtain risk factors for mortality. Covariance diagnosis was used to assess the presence of multicollinearity between variables. The area under the curve (AUC) of the receiver operating characteristic (ROC) was used to assess the predictive value of relevant risk factors for death 28 days after the diagnosis of moderate-to-severe PARDS. A *p-*value <0.05 was considered statistically significant for all the tests.

Latent class analysis (LCA) was performed using Mplus 7.0 to estimate the model, it based on the full information maximum likelihood method and allows the use of all data from all patients, including those with some missing data. The evaluation indices (10) for model fitting were as follows: (i) the Akaike information criterion (AIC) and Bayesian information criterion (BIC), with smaller values of AIC and BIC indicating a better-fitting model; (ii) the Lo–Mendell–Rubin test (LMR) to test whether the number of classes provided improved model fit compared to the model using one fewer class; (iii) entropy to evaluate the accuracy of model classification, with a range of 0–1, where values ≥0.8 are generally considered a sign of a useful model. The number of classes was ultimately decided through the combination of model fit indices. The survival probability of patients with different subphenotypes of moderate-to-severe PARDS in the ICU was determined using Kaplan–Meier survival curves.

## Results

**Figure 1:**
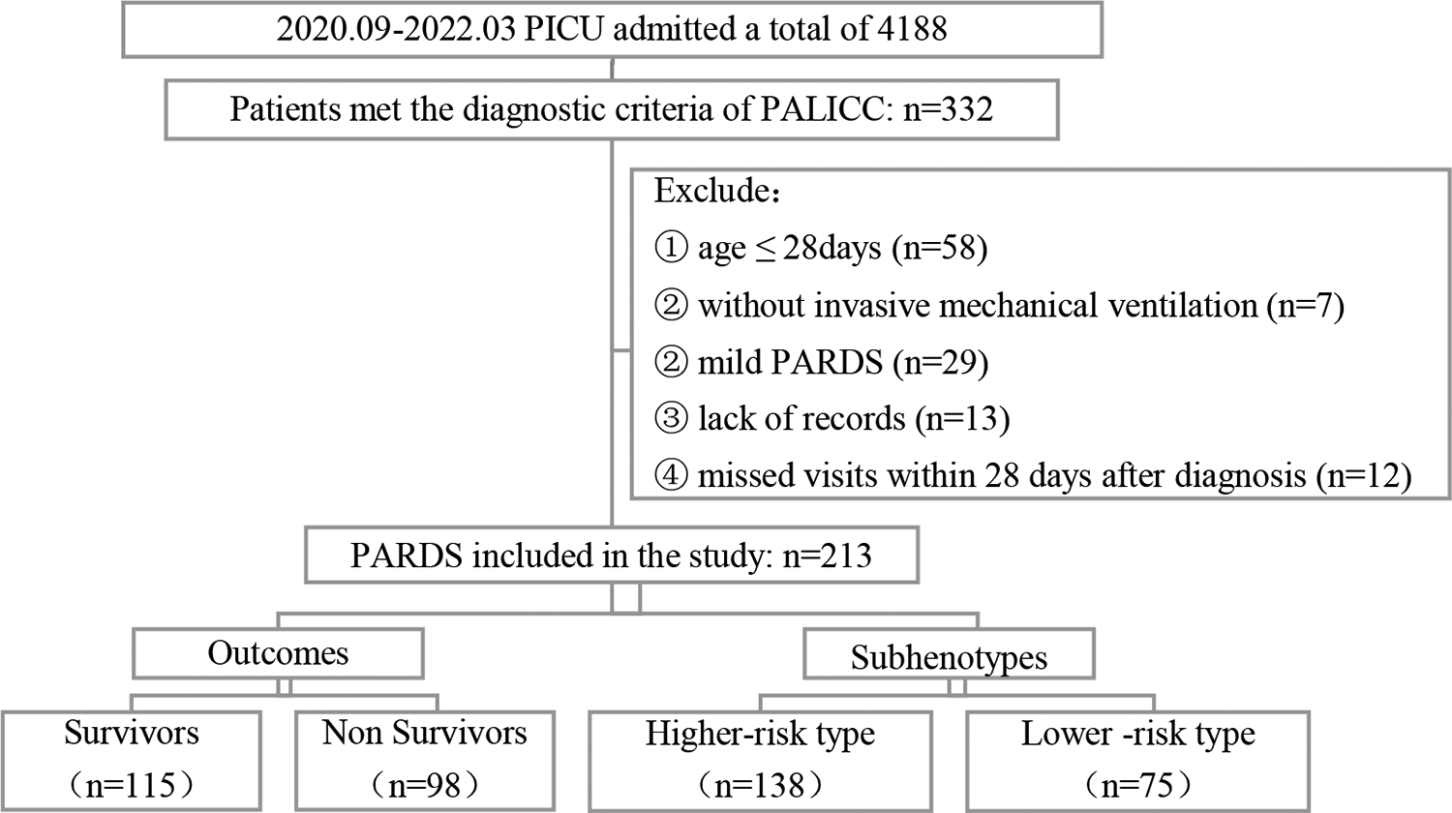
Flow chart of screening. PICU: Pediatric Intensive Care Unit; PALICC, Pediatric Acute Lung Injury Consensus Conference; PARDS: Pediatric Acute Respiratory Distress Syndrome.

A total of 231 children were included in the study according to the inclusion and exclusion criteria; the screening process is shown in Figure 1.

### Demographic and baseline information

The age of the patients ranged from 30 days to 16 years, and 125 (58.7%) of them were male. A total of 98 (46.0%) patients died 28 days after diagnosis of PARDS (Figure 1), with no significant differences in mortality by gender; pneumonia or lower-respiratory-tract infection (93.9%) and sepsis (20.7%) were the most common risk factors for PARDS. Furthermore, 63.3% of children had comorbidities before the onset of PARDS, with the most common comorbidity being genetic disorders (35.7%), followed by hematologic or immunologic diseases (29.1%).

All patients were divided into survivors and non-survivors according to prognosis 28 days after PARDS diagnosis, and their baseline data were compared (Table 1), with statistically significant differences observed in PIM3 score, PELOD2 score, oxygenation index (OI), division, sepsis, and presence and type of comorbidities between the two groups.

**Table 1:** Demographic and baseline information.

	Survivors (*n* = 115)	Non-survivors (*n* = 98)	*χ* ²/Z	*P*
Gender			0.492	0.483
Male, *n* (%)	70 (60.9)	55 (56.1)	/	/
Female, *n* (%)	45 (39.1)	43 (43.9)	/	/
Age, median and quartile, y	1.4 (0.6, 4.0)	1.7 (0.5, 6.1)	−0.94	0.347
Weight, median and quartile, kg	9.5 (6.5, 17.5)	10.0 (7.0, 18.4)	−0.749	0.454
WAZ, median and quartile	−1.25 (−2.59, −0.11)	−0.92 (−2.53, −0.10)	−0.656	0.512
PIM3, median and quartile	7.1 (3.0, 46.4)	18.5 (5.9, 88.0)	−3.403	0.001
PELOD2, median and quartile	4 (1, 10)	9 (5, 13)	−4.358	<0.001
OI, median and quartile	11.43 (8.65, 20.0)	16.55 (9.97, 24.00)	−2.426	0.015
Sever PARDS, *n* (%)	40 (34.8)	52 (53.1)	7.205	0.007
PARDS Risk Factors				
Pneumonia, *n* (%)	107 (93.0)	93 (94.9)	0.318	0.573
Aspiration, *n* (%)	1 (0.9)	0 (0)	/	1.000
Drowning, *n* (%)	4 (3.5)	2 (2.0)	/	0.689
Sepsis, *n* (%)	11 (9.6)	31 (31.6)	16.277	<0.001
Trauma, *n* (%)	1 (0.9)	0 (0)	/	1.000
Pancreatitis, *n* (%)	0 (0)	1 (1.0)	/	0.460
Other, *n* (%)	8 (7.0)	4 (4.1)	0.823	0.364
Comorbidities				
Any Comorbidity, *n* (%)	54 (47.0)	81 (82.7)	29.049	<0.001
Neuromuscular Disease, *n* (%)	10 (8.7)	14 (14.3)	1.654	0.198
Cardiovascular Disease, *n* (%)	23 (20.0)	23 (23.5)	0.376	0.540
Respiratory Disease, *n* (%)	10 (8.7)	7 (7.1)	0.174	0.677
Kidney Disease, *n* (%)	9 (7.8)	3 (3.1)	2.260	0.133
Digestive Disease, *n* (%)	5 (4.3)	7 (7.1)	0.777	0.378
Hematologic or Immunologic Diseases, *n* (%)	20 (17.4)	43 (43.9)	17.820	<0.001
Metabolic Disease, *n* (%)	7 (6.1)	7 (7.1)	0.095	0.757
Hereditary Disease, *n* (%)	35 (30.4)	41 (41.8)	2.997	0.083
Tumor, *n* (%)	2 (1.7)	5 (5.1)	1.883	0.170
Laboratory Examination				
Lactate, mmol/L	1.0 (0.6, 1.7)	1.0 (0.6, 2.0)	−0.398	0.691
pH	7.40 (7.32, 7.46)	7.36 (7.27, 7.46)	−1.651	0.099
PaO_2_, mmHg	64.0 (53.0, 80.9)	62.0 (49.8, 86.1)	−0.493	0.622
PaCO_2_, mmHg	43.0 (35.3, 7.53)	44.0 (36.0, 55.2)	−0.718	0.473
Positive Blood Pathogen, *n* (%)	11 (9.6)	13 (13.3)	0.725	0.395
Positive Sputum Pathogen, *n* (%)	48 (41.7)	33 (33.7)	1.461	0.227

WAZ, weight-for-age z-score; PIM3, Pediatric Index of Mortality 3 score; PELOD2, Pediatric Logistic Organ Dysfunction 2 score; OI, oxygenation index; PARDS, pediatric acute respiratory distress syndrome; PaO2, partial pressure of oxygen of arterial blood; PaCO2: partial pressure of carbon dioxide of arterial blood.

There was no statistically significant difference in the rate of positive pathogens between the two groups of children. A total of 81 (38.1%) had positive sputum pathogen, including 53 (65.4%) bacterial and 34 (42.0%) viral infections, with adenovirus infection (21, 25.9%) being the most frequent, followed by *Acinetobacter baumannii* infection (20, 24.7%). Furthermore, 24 of 213 patients (11.3%) were positive for blood pathogens, including 20 bacterial infections, three fungal infections, and only one combined bacterial and fungal infection; the incidence of Gram-negative bacterial infections (15, 65.2%) was highest. Six cases of cytomegalovirus and three cases of Epstein–Barr virus positivity were also detected by polymerase chain reaction (PCR) assay.

### Treatment information and outcome

Continuous renal replacement therapy (CRRT) was used more in non-survivors. The duration of invasive mechanical ventilation, length of ICU stay, and hospitalization time of non-survivors were shorter, but the incidence of multiple organ dysfunction syndrome (MODS) was higher, especially the incidence of organ dysfunction in the circulatory, urinary, and hematological systems (Table 2).

**Table 2:** Treatment information and outcomes.

	Survivors (*n* = 115)	Non-survivors (*n* = 98)	χ ²/Z	*P*
Re-intubation, *n* (%)	12 (10.4)	4 (4.1)	3.074	0.080
HFOV, *n* (%)	29 (25.2)	34 (34.7)	2.281	0.131
Pulmonary Vasodilators, *n* (%)	20 (17.4)	15 (15.3)	0.168	0.682
Neuromuscular Blocking Agents, *n* (%)	106 (92.2)	87 (88.8)	0.718	0.397
Prone Ventilation, *n* (%)	21 (18.3)	17 (17.3)	0.030	0.862
Systemic Steroid, *n* (%)	58 (50.4)	37 (37.8)	3.443	0.064
Surfactants, *n* (%)	2 (1.7)	5 (5.1)	1.883	0.170
*β*-Receptor Agonist, *n* (%)	43 (37.4)	47 (48.0)	2.442	0.120
Diuretic, *n* (%)	104 (90.4)	85 (86.7)	0.725	0.395
Blood transfusion, *n* (%)	66 (57.4)	66 (67.3)	2.225	0.136
CRRT, *n* (%)	4 (3.5)	12 (12.2)	5.853	0.016
ECMO, *n* (%)	2 (1.7)	3 (3.1)	0.403	0.525
Intubation time, median and quartile, d	11 (6, 16)	8 (3, 15)	−2.415	0.016
Days of PICU, median and quartile, d	15 (9, 23)	9 (4, 18)	−4.133	<0.001
Hospital stays, median and quartile, d	23 (14, 37)	18 (9, 27)	−3.327	0.001
MODS, *n* (%)	14 (12.2)	35 (35.7)	16.554	<0.001
Circulatory Failure, *n* (%)	12 (10.4)	22 (22.4)	5.693	0.017
Nervous system failure, *n* (%)	16 (13.9)	7 (7.1)	2.518	0.113
Liver Failure, *n* (%)	10 (8.7)	13 (13.3)	1.147	0.284
Urinary Failure, *n* (%)	3 (2.6)	16 (16.9)	12.255	<0.001
Hematological Systems Failure, *n* (%)	3 (2.6)	13 (13.3)	8.649	0.003

HFOV, high frequency oscillation; CRRT, continuous renal replacement therapy; ECMO, extracorporeal membrane oxygenation; MODS, multiple organ dysfunction syndrome.

### Analysis of risk factors for death

Univariate and multivariate analyses through binary logistic regression were performed to compare the patients in the two groups (Tables 3, 4). Multivariable analysis showed that PELOD2, sepsis, any comorbidity, comorbid hematologic or immunologic diseases, IAG, and level of organ failure were associated with worse outcomes, with tolerances >0.1 and variance inflation factor (VIF) <10, suggesting that these factors were not multiple covariates (Table 4).

**Table 3:** Univariate logistic regression models.

	OR	95CI	*P*
Gender, male	1.216	0.704–2.102	0.483
WAZ	1.064	0.885–1.280	0.509
PIM3	2.899	1.362–6.173	0.006
PELOD2	1.112	1.055–1.172	<0.001
OI	1.026	1.001–1.052	0.042
Pneumonia	1.391	0.440–4.398	0.575
Drowning	0.578	0.104–3.226	0.532
Sepsis	4.374	2.060–9.291	<0.001
Any Comorbidity	5.382	2.843–10.191	<0.001
Neuromuscular Disease	1.750	0.740–4.139	0.203
Cardiovascular Disease	1.227	0.638–2.358	0.540
Respiratory Disease	0.808	0.295–2.208	0.677
Kidney Disease	0.372	0.098–1.414	0.147
Digestive Disease	1.692	0.520–5.512	0.383
Hematologic or Immunologic Diseases	3.714	1.986–6.944	<0.001
Metabolic Disease	1.187	0.401–3.509	0.757
Hereditary Disease	1.644	0.935–2.892	0.084
Tumor	3.038	0.576–16.018	0.190
Lactate	1.103	0.974–1.249	0.123
pH	0.174	0.022–1.348	0.094
PaO_2_	1.001	0.990–1.012	0.897
PaCO_2_	0.999	0.985–1.014	0.943
Blood Culture, Positive	1.446	0.616–3.392	0.397
Sputum Culture, Positive	0.709	0.405–1.240	0.228
IAG	2.676	1.808–4.235	<0.001
Re-intubation	0.365	0.114–1.172	0.09
HFOV	1.575	0.872–2.847	0.132
Pulmonary Vasodilators	0.858	0.413–1.784	0.682
Neuromuscular Blocking Agents	0.672	0.266–1.694	0.399
Prone Ventilation	0.939	0.464–1.902	0.862
Systemic Steroid	0.596	0.345–1.031	0.064
Surfactants	3.038	0.576–16.018	0.190
βReceptor Agonist	1.543	0.893–2.668	0.120
Diuretic	0.692	0.295–1.622	0.397
Blood transfusion	1.531	0.874–2.684	0.137
CRRT	5.209	1.425–19.038	0.013
ECMO	1.784	0.292–10.901	0.531
Level of Organ Failure	1.456	1.083–1.957	0.013

WAZ, weight-for-age z-score; PIM3, Pediatric Index of Mortality 3 score; PELOD2, Pediatric Logistic Organ Dysfunction 2 score; OI, oxygenation Index; PaO2, partial pressure of oxygen of arterial blood; PaCO2: partial pressure of carbon dioxide of arterial blood; IAG, infiltration area grade; HFOV, high frequency oscillation; CRRT, continuous renal replacement therapy; ECMO, extracorporeal membrane oxygenation.

**Table 4:** Multivariate logistic regression models.

	OR	95CI	*P*	Tolerance	VIF
PELOD2	1.082	1.004–1.166	0.04	0.583	1.716
Sepsis	4.234	1.773–10.111	0.001	0.947	1.056
Any Comorbidity	3.437	1.489–7.936	0.004	0.677	1.478
Hematologic or Immunologic Diseases	2.013	0.926–4.376	0.077	0.768	1.303
Level of Organ Failure	1.617	1.113–2.313	0.009	0.864	1.158
IAG	1.98	1.028–3.813	0.041	0.517	1.933
PIM3	0.375	0.094–1.498	0.165	0.397	2.516
OI	1.027	0.997–1.058	0.083	0.925	1.081
CRRT	1.776	0.452–6.979	0.411	0.93	1.075

PELOD2, Pediatric Logistic Organ Dysfunction 2 score; PIM3, Pediatric Index of Mortality 3 score; VIF, variance inflation factor; OI, oxygenation Index; CRRT, continuous renal replacement therapy; IAG, infiltration area grade.

### Prediction of short-term prognosis

**Figure 2:**
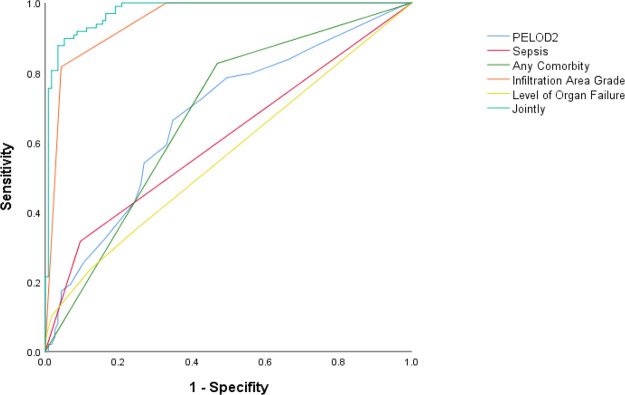
ROC curves of various indicators predicting death 28 days after diagnosis.

PELOD2, sepsis, any comorbidity, IAG, and level of organ failure were included in the multivariable logistic regression, and the predicted probabilities obtained were used as the combined values for joint prediction. Then, the ROC curves were plotted (Figure 2), revealing that the AUC of the joint indicators predicting death 28 days after diagnosis was largest (Table 5), with a sensitivity of 89.79% and a specificity of 94.78%.

**Table 5:** AUC of various indicators predicting death 28 days after diagnosis.

	AUC	95%CI	*p*
PELOD2	0.672	0.600–0.745	<0.001
Sepsis	0.610	0.533–0.687	0.006
Any Comorbidity	0.678	0.606–0.751	<0.001
IAG	0.948	0.919–0.977	0.015
Level of Organ Failure	0.564	0.486–0.642	0.110
Joint Prediction	0.976	0.958–0.993	<0.001

PELOD2, Pediatric Logistic Organ Dysfunction 2 score; IAG, infiltration area grade.

### Latent class analysis

A latent class analysis was performed with 1–5 classes. It was found that the entropy was higher than 0.8 in all models, suggesting that the models were all accurate; the value of BIC was the smallest in the three-class model, while the AIC was the smallest when the number of model categories was five. The value of the LMR test was lower than 0.05 only when the number of classes was two or three. Considering all indicators, the analysis of latent class models suggested that the two-class model and three-class model were best (Table 6). We chose a two-class model for simplicity, referring to the two classes as phenotypes 1 and 2. According to the model, 131 cases were assigned to phenotype 1, and 82 cases were assigned to phenotype 2 (Figure 1).

**Table 6:** Selection of the best model.

Number of classes	AIC	BIC	Entropy	LMR	N1	N2	N3	N4	N5
2	3091.323	3182.078	0.883	<0.001	131	82			
3	3042.222	3180.035	0.976	0.039	74	69	70		
4	3021.482	3206.353	0.872	0.103	24	48	74	67	
5	3017.068	3248.998	0.869	0.450	68	43	31	28	43

AIC, Akaike information criterion; BIC, Bayesian information criterion; LMR, Lo Mendell-Rubin.

**Figure 3:**
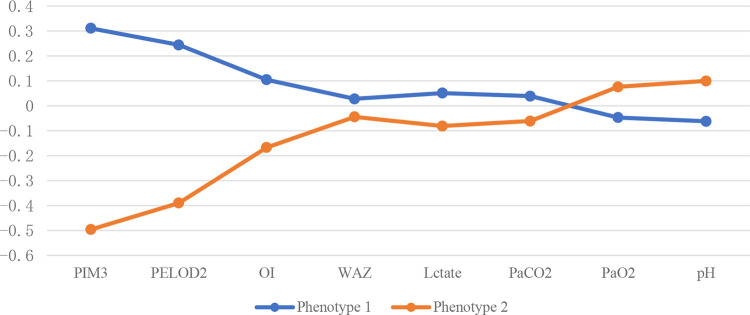
Continuous variables of different subphenotypes. PELOD2, Pediatric Logistic Organ Dysfunction 2 score; PIM3, Pediatric Index of Mortality 3 score; OI, oxygenation Index; WAZ, weight-for-age z-score.

**Figure 4:**
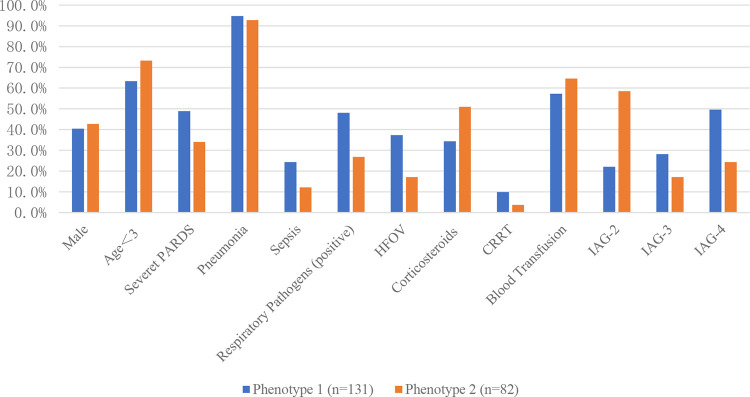
Categorical variables of different subphenotypes. HFOV, high frequency oscillation; CRRT, continuous renal replacement therapy; IAG, infiltration area grade.

The standardized values of continuous variables are shown in Figure 3, and the analysis of categorical variables is shown in Figure 4, which differed between the two phenotypes. The following indicators presented statistically significant differences: PIM3 score (*p* < 0.01), PELOD2 score (*p* < 0.01), sepsis (24.4% vs. 12.2%, *p* = 0.029), positive respiratory pathogens (48.1% vs. 26.8%, *p* = 0.002), and IAG distribution (*p* < 0.001). Children with IAG-4 were more frequent in the higher-risk group, while those with IAG-2 were more frequent in the lower-risk group. For another, the application of high-frequency oscillation ventilation and corticosteroids was also significantly different (37.4% vs. 17.1%, *p* = 0.002 and 34.4% vs. 51.0%, *p* < 0.01, respectively). Combining these differences, phenotype 1 was denoted as the higher-risk group, and phenotype 2 was denoted as the lower-risk group. Their clinical outcomes were also distinct, with a higher mortality in patients of phenotype 1 (61.1% vs. 22.0%, *p* < 0.01).

**Figure 5:**
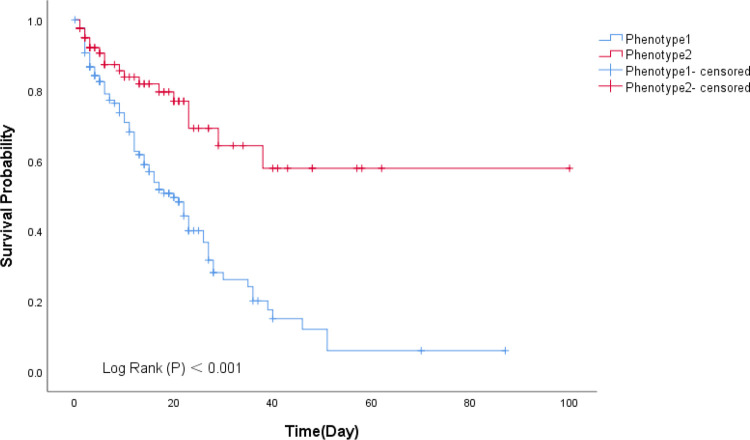
Kaplan-Meier curves of different subphenotypes.

The survival analysis showed the probability of survival decreased significantly at 0 to 40 days for the phenotype 1 than phenotype 2 (*p* < 0.001), and this difference became more prominent over time (Figure 5).

## Discussion

ARDS is a syndrome caused by multiple diseases that can lead to refractory and life-threatening hypoxemia. Despite decades of research on possible treatment strategies, the mortality rate of moderate-to-severe PARDS remains high. In Asia, the mortality rate of patients with PARDS ranges from 44% to 75%, in contrast to the lower rates reported in Europe and Australia/New Zealand (17%–35%) (3, 11).

After the Pediatric Acute Lung Injury Consensus Conference developed a specific definition of PARDS in 2015 (9), the Asian Pediatric Intensive Care Network published the latest research data (3) that Chinese and Southeast Asian children with PARDS had an overall PICU mortality rate of 30.3% and a 100 day mortality rate of 39.7%. In addition, there has been a significant decrease in PARDS mortality in the last two decades in Western countries but not in Asia. This study showed a mortality rate of 46.0% of moderate-to-severe PARDS in the largest PICU in southwest China. This is relatively high since the study focused on moderate-to-severe patients. The differences in the characteristics of the study population, resource combination, case distribution, socioeconomic conditions, and management strategies may also contribute to mortality differences. Improved treatment and management strategies may ameliorate the prognosis of patients with PARDS.

In our prospective observational study of 213 patients, we found that pneumonia and sepsis were the main etiology of moderate-to-severe PARDS, and sepsis as a major risk factor led to the deterioration of clinical outcomes. The difficulty of extubation, slow recovery from lung injury, and poor clinical outcomes in patients with sepsis-related moderate-to-severe PARDS (12) are linked with the pathological process, in which the inflammatory response initially helps to clear invading pathogens, but later, pathogens cause host defense dysfunction by inducing apoptosis of immune effector cells in order to seek an advantage (13). In this way, the high inflammatory state evolves into a low inflammatory state, eventually leading to immunosuppression and physiological destruction. It has been established that, in addition to immune organs, the immune function of other organs such as the lung is also obviously altered during the development of sepsis (14). Therefore, in the treatment of sepsis-related moderate-to-severe ARDS, timely and rational antibiotic administration and target immunotherapy are very important (14), in addition to the optimal ventilation strategy according to the lung condition. There are also preclinical studies confirming the benefits of aspirin (15) and mesenchymal stem cells (16) in sepsis-related moderate-to-severe ARDS, but their effectiveness has not yet been clarified through clinical trials.

The presence of comorbidities is also a risk factor contributing to the poor prognosis of patients with moderate-to-severe PARDS, with even worse outcomes in those with comorbid hematologic or immunologic diseases compared to other comorbidities, which indicates the adverse effects of an immunosuppressed state on moderate-to-severe PARDS. As a systemic inflammatory syndrome, ARDS is not limited to the lung; thus, the underlying disease or the immunosuppressive state caused by therapy may lead to the dissemination of inflammatory mediators to other organs and systems and eventually lead to the occurrence of MODS, the main reason for the high mortality rate in the late stages of ARDS (17). MODS is recognized as a common risk factor for death in general ICU patients (18, 19), which further leads to “immune paralysis” (20). The processes of both MODS and immune injury are mutually reinforcing. As PARDS gets worse and the level of organ failure increases, the risk of adverse outcomes increases. Another risk factor for death in our study was an increase in PELOD2 score, which is used to assess the severity of organ dysfunction, including central nervous, cardiovascular, renal, respiratory, and hematologic systems. A higher score indicates more severe organ dysfunction and a higher risk of death 28 days after diagnosis. Several studies have shown that the PELOD2 score performs well in predicting in-hospital mortality and even long-term prognosis (21–25).

The diagnosis criteria of ARDS in adults require diffused infiltration of the bilateral lung in chest x-ray, in contrast to the diagnostic criteria in children (9). This does not mean that changes in chest radiographs are meaningless, since there are no reliable clinical data so far. We defined the IAG from the chest x-ray of the patients with moderate-to-severe PARDS to describe the severity the lesion of lung, and IAG was identified as another vital risk factor for death as expected. The diagnosis of PARDS depends in part on identifying characteristic radiographic abnormalities; hence, interpretation of a radiological investigation is important. We found that all patients were actually assessed higher than IAG-2. A more severe lesion of the lung led to a higher risk of death. This affirmed the significance of follow-up chest radiographs in disease progression.

After plotting the ROC curves, it was seen that the AUC of the combination of PELOD2, sepsis, any comorbidity, IAG, and level of organ failure to predict prognosis 28 days after diagnosis in patients with moderate-to-severe PARDS was significantly larger than each indicator alone, while OI at the beginning of the disease was not a decisive predictor for PARDS. Accordingly, this may provide a reference for clinical assessment of short-term prognosis and individualized treatment plans for patients with moderate-to-severe PARDS.

Most of the infections in patients with moderate-to-severe PARDS in this study were caused by bacteria, predominantly Gram-negative bacteria, with *Acinetobacter baumannii* accounting for the largest proportion. This is due to the fact that patients with moderate-to-severe PARDS may be in a state of immunosuppression (26), and invasive treatment strategies such as tracheal intubation tend to lead to a relative increase in nosocomial infections. Ventilator-associated tracheobronchitis due to pathogenic colonization prolongs the duration of mechanical ventilation and ICU stay (27), and inappropriate treatment increases the risk of developing ventilator-associated pneumonia (28); thus, timely anti-infective treatment is crucial. The association between positive pathogens and poorer outcomes was not reflected in this study, which is related to the fact that the study population was PICU patients, who were generally treated prior to PICU admission and were subjected to relatively aggressive anti-infective treatment in the early course of the disease, resulting in a high rate of false-negative pathogenic tests after admission. Viruses are also a major cause of infection in patients with moderate-to-severe PARDS, and common respiratory viruses include adenovirus and respiratory syncytial virus, in addition to some latent viruses such as cytomegalovirus that are reactivated in an immunosuppressed state and may prolong hospitalization and increase the risk of death (29). Some studies have claimed that viral pneumonia due to RSV and influenza viruses may have better outcomes compared to other viruses (30, 31); however, we did not observe significant differences, which may have stemmed from virus subtypes, study period, sample size, and possibly the heterogeneity of PARDS patients themselves.

To further analyze the heterogeneity of moderate-to-severe PARDS, two subphenotypes with different baseline characteristics and significantly different clinical outcomes were identified in this study by latent class analysis. Phenotype 1, characterized by higher PIM3 scores and PELOD2 scores, had a poorer clinical outcome and more risk factors for death, including more patients with sepsis, inherited metabolic diseases, positive respiratory pathogens, and higher IAG. Accordingly, phenotype 1 was characterized as a high-risk type, while phenotype 2 was characterized as a lower-risk type. The difference in prognosis based on these indicators is a novel finding.

The treatment of all included patients was based on invasive mechanical ventilation combined with symptomatic supportive therapy (such as medications, nutritional support, and fluid restriction) and advanced life support (such as continuous renal replacement therapy (CRRT) and extracorporeal membrane lung oxygenation (ECMO)) as needed. We found significant differences in the application of corticosteroids in the two subphenotypes, because the use of corticosteroids in the higher-risk group required greater caution. They were not recommended as routine therapy in PARDS in the census of PALICC (9). However, as a commonly used therapy in PICU, further research in the future into specific patient populations of PARDS for corticosteroid therapy, as well as the dose and route of treatment, is quite necessary. We also observed that more higher-risk types used high-frequency ventilation than lower-risk types, due to the worse baseline oxygenation in higher-risk types. Some studies have suggested that the use of HFOV within the first week after diagnosis increases the mortality risk (11). However, the present study failed to confirm this, which may be related to the different distribution of PARDS subphenotypes in different studies. The application of CRRT in higher-risk types is relatively greater, which may stem from the relatively greater acute kidney injury in patients of higher-risk phenotype. The need for CRRT reflects the rapid progression and severity of renal dysfunction in higher-risk patients, with deteriorating clinical status and eventual death despite the provision of strong advanced support. PALICC suggested that ECMO may be considered in patients with respiratory failure due to PARDS (9), but there are no clinical criteria to judge the more suitable population. Only five children in this study received ECMO, and there was no significant difference in prognosis.

Two phenotypes were also obtained in ARDS studies (32), one of which was characterized by a high inflammatory response, shock, and metabolic acidosis. Their differentiation was mainly based on multiple biological markers. Different phenotypes respond differently to multiple therapeutic strategies including high positive end-expiratory pressure (PEEP) (32), conservative fluid management (33), and statins (34, 35). Some similar biological features have been observed in other critically ill patients without ARDS, suggesting that the phenotypes of ARDS are generalizable. Distinct groups may have similar systemic immune processes, and the study of phenotypes may provide help in the treatment of more critically ill patients (36).

As a single-center study, the diagnosis and treatment of moderate-to-severe PARDS in the study were uniformly and strictly performed according to the PALICC criteria; thus, there was less heterogeneity, and the results were quite reliable. However, there were some limitations. First, this study was a single-center observational study, which needs to be revalidated with data from a large multicenter sample. Second, the duration of our study was relatively short, and more conclusions may be obtained by extending the observation time. In addition, more clinical indicators and biological markers could be included, so as to increase the accuracy of the phenotypic characterization of moderate-to-severe PARDS. Since moderate-to-severe PARDS is the end stage of many diverse etiologies that have unique pathophysiologic processes, it is difficult to find a universally effective treatment. Further clarity and certainty of these conclusions are needed in the future with additional large-scale randomized controlled trials.

## Data Availability

The original contributions presented in the study are publicly available. This data can be found here: https://data.mendeley.com/datasets/4j5mh9n7j7.
